# *Ex Vivo* Expansion of Human Hematopoietic Stem Cells by Garcinol, a Potent Inhibitor of Histone Acetyltransferase

**DOI:** 10.1371/journal.pone.0024298

**Published:** 2011-09-12

**Authors:** Taito Nishino, Changshan Wang, Makiko Mochizuki-Kashio, Mitsujiro Osawa, Hiromitsu Nakauchi, Atsushi Iwama

**Affiliations:** 1 Research Planning Department, Nissan Chemical Industries, Tokyo, Japan; 2 Department of Cellular and Molecular Medicine, Graduate School of Medicine, Chiba University, Chiba, Japan; 3 Division of Stem Cell Therapy, Center for Stem Cell Biology and Regenerative Medicine, Institute of Medical Science, University of Tokyo, Tokyo, Japan; 4 Japan Science and Technology Agency, CREST, Tokyo, Japan; 5 ERATO, Tokyo, Japan; University of Medicine and Dentistry of New Jersey, United States of America

## Abstract

**Background:**

Human cord blood (hCB) is the main source of hematopoietic stem and progenitor cells (HSCs/PCs) for transplantation. Efforts to overcome relative shortages of HSCs/PCs have led to technologies to expand HSCs/PCs *ex vivo*. However, methods suitable for clinical practice have yet to be fully established.

**Methodology/Principal Findings:**

In this study, we screened biologically active natural products for activity to promote expansion of hCB HSCs/PCs *ex vivo*, and identified Garcinol, a plant-derived histone acetyltransferase (HAT) inhibitor, as a novel stimulator of hCB HSC/PC expansion. During a 7-day culture of CD34^+^CD38^–^ HSCs supplemented with stem cell factor and thrombopoietin, Garcinol increased numbers of CD34^+^CD38^–^ HSCs/PCs more than 4.5-fold and Isogarcinol, a derivative of Garcinol, 7.4-fold. Furthermore, during a 7-day culture of CD34^+^ HSCs/PCs, Garcinol expanded the number of SCID-repopulating cells (SRCs) 2.5-fold. We also demonstrated that the capacity of Garcinol and its derivatives to expand HSCs/PCs was closely correlated with their inhibitory effect on HAT. The Garcinol derivatives which expanded HSCs/PCs inhibited the HAT activity and acetylation of histones, while inactive derivatives did not.

**Conclusions/Significance:**

Our findings identify Garcinol as the first natural product acting on HSCs/PCs and suggest the inhibition of HAT to be an alternative approach for manipulating HSCs/PCs.

## Introduction

Hematopoietic stem cells (HSCs) have been applied to the treatment of a wide variety of blood disorders through HSC transplantations and gene therapy [1]–[3]. Related as well as unrelated human cord blood (hCB) has emerged as a source of HSCs and the use of these cells is increasing because of the low risk of graft-versus-host disease and facile procurement [4]. To date, over 10,000 hCB transplantations have been conducted [5]. However, the widespread application of hCB is limited by relatively low numbers of HSCs, resulting in a significant delay in hematopoietic recovery and increased transplant-related mortality. Thus approaches that can overcome low cell doses and delayed engraftment are of great interest. The cotransplantation of two CB units from different donors, which increases the available cell dose, has been used. Alternatively, numerous attempts have been made to expand hCB HSCs *ex vivo* [6]–[11]. The majority of cell culture systems have exploited protein-factor mixtures, including stem cell factor (SCF), thrombopoietin (TPO), fms-like tyrosine kinase 3 ligand (FL), a complex of interleukin 6 (IL-6) and soluble IL-6 receptor (IL-6/sIL-6R), the Notch ligand Delta1, Angiopoietin-like proteins, and Pleiotrophin. Notably, Delaney and colleagues reported that transplantation with Notch-mediated expansion *ex vivo* resulted in faster neutrophil engraftment compared to a control group receiving uncultured hCB [8]. However, additional clinical studies will be required to confirm the enhanced kinetics of engraftment in humans, and identification of the cell signaling that governs the self-renewal of HSCs is needed to improve existing methods of hCB HSC expansion *ex vivo*. It could also be pointed out that protein-factor combinations have proven to be neither cost-effective nor readily available.

Small-molecule compounds (SMCs), which comprise natural and chemically synthesized products, have played a pivotal role in molecular biology and pharmaceutical therapy. The use of SMCs has also facilitated elucidation of the signaling pathways that control stemness and been applied to HSC expansion *ex vivo* [12]–[16]. The method using tetraethylenepentamine, a synthetic copper chelator, which expands hCB CD34^+^ cells and increases their potential for engraftment in immunodeficient mice, has shown feasibility in a Phase I/II study [15]. Boitano and colleagues reported that a chemically synthesized purine derivative induced hCB HSC expansion in culture by antagonizing the aryl hydrocarbon receptor [16]. We also reported that activation of the human thrombopoietin receptor by a small-molecule agonist promoted expansion of hCB HSCs [17]. Nonetheless, there is a need to identify more efficient SMCs and to design better compounds in terms of efficacy and safety for clinical use.

Here, in a search for biologically active natural products that may activate signals required for HSC expansion, we screened natural products for effects on hCB CD34^+^CD38^–^ cells, which are reported to be primitive hematopoietic stem and progenitor cells (HSCs/PCs) [18], [19]. We found that Garcinol, a benzophenone derivative originally isolated from Garcinia indica [20], [21], expands HSCs/PCs through an inhibitory effect on HAT. This is the first report of a small-molecule HAT inhibitor promoting HSC expansion *ex vivo*.

## Results

### Garcinol and its derivative expand human hematopoietic progenitors

**Figure 1:**
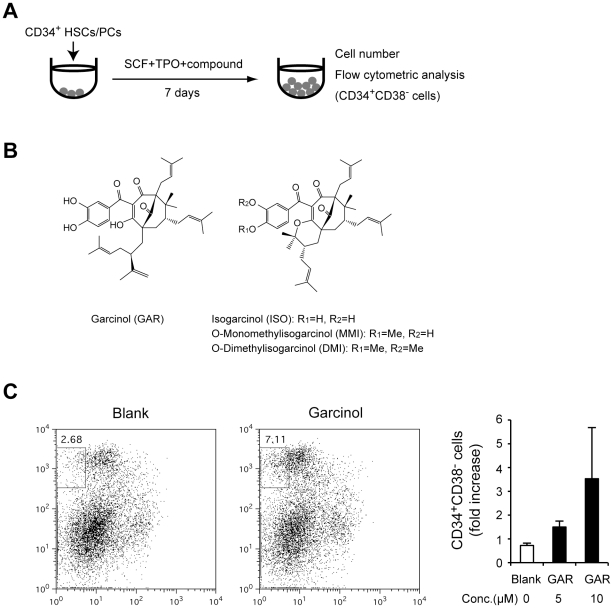
Identification of Garcinol as a natural product biologically active on HSCs/PCs. A. Experimental design for the screening of natural compounds acting on HSCs/PCs. **B.** Chemical structure of Garcinol and its derivatives. **C.** Garcinol expands CD34^+^CD38^–^ cell numbers. hCB CD34^+^ cells were cultured in StemSpan SFEM medium with Garcinol or the same volume of DMSO (blank) in the presence of 20 ng/mL of hrTPO and 100 ng/mL of hrSCF. At day 7 of culture, the cells were analyzed by FACS for CD34 and CD38 expression (left panel). The numbers of CD34^+^CD38^–^ cells relative to the blank are indicated [mean±standard error of the mean (SEM), n = 3]. *p<0.05.

**Figure 2:**
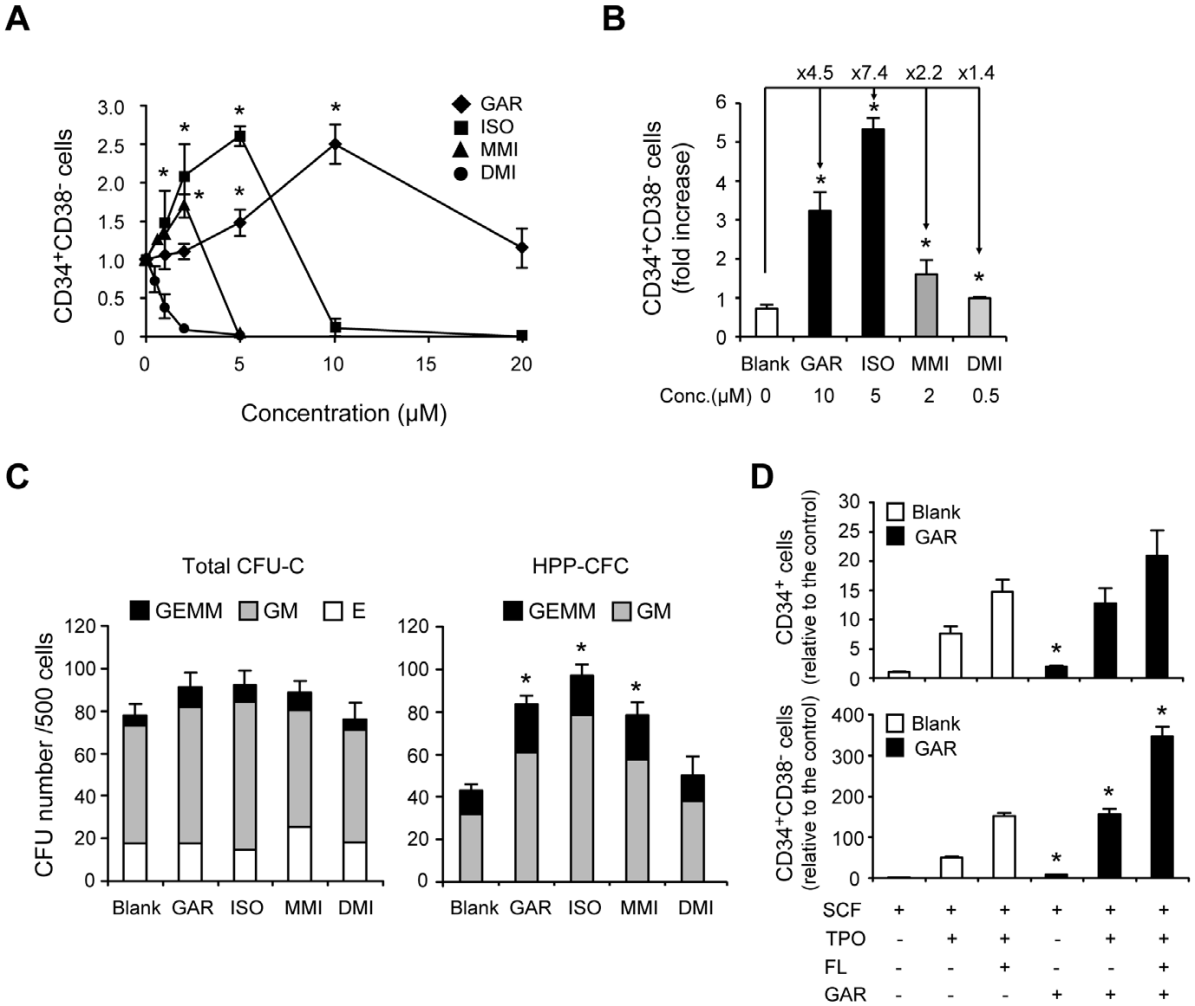
Garcinol efficiently expands numbers of human CD34^+^CD38^–^ cells and colony-forming cells. **A.** The effects of Garcinol derivatives on hCB CD34^+^ HSCs/PCs. hCB CD34^+^ cells were cultured in StemSpan SFEM medium with Garcinol derivatives or the same volume of DMSO (blank) in the presence of 20 ng/mL of hrTPO and 100 ng/mL of hrSCF for 7 days. Cultured cells were counted with a hemocytometer (trypan blue exclusion) and analyzed by FACS for CD34 and CD38 expression. The numbers of CD34^+^CD38^–^ cells relative to the blank are indicated (mean±SEM, n = 3). *p<0.05. **B.** The effects of Garcinol derivatives on hCB CD34^+^CD38^–^ HSCs. CD34^+^CD38^–^ cells were isolated from hCB cells by FACSAria and cultured as in **A**. Bars represent the fold-increase in the number of CD34^+^CD38^–^ cells in the 7-day cultures compared with the initial number of CD34^+^CD38^–^ cells (mean±SEM, n = 3). *p<0.05. **C.** The effects of Garcinol derivatives on colony-forming cells. The 7-day cultures of hCB CD34^+^ cells treated with Garcinol derivatives or the same volume of DMSO (blank) were plated in Methocult GF H4435 methylcellulose medium (500 cells/plate), and colonies were counted after 12 days. Bars represent the number of colony-forming units in culture (CFU-C) and HPP-CFCs per 500 cultured cells (mean±SEM, n = 3). *p<0.05. **D.** The effects of Garcinol derivatives on HSCs/PCs in the presence of multiple cytokines. hCB CD34^+^ cells were cultured with Garcinol (10 µM) or the same volume of DMSO (blank) in the presence of SCF (100 ng/mL) alone, SCF and TPO (20 ng/mL), or SCF, TPO, and FL (50 ng/mL) for 7 days. The numbers of CD34^+^CD38^–^ cells relative to that in the control culture supplemented with SCF only are indicated (mean±SEM, n = 3). The number of CD34^+^CD38^–^ cells in the control culture was arbitrarily set to 1. *p<0.05.

To identify biologically-active natural products that act on HSCs/PCs *ex vivo*, we cultured hCB CD34^+^ HSCs/PCs with natural products in the presence of stem cell factor (SCF) and thrombopoietin (TPO) for 7 days and examined the number of CD34^+^CD38^-^ HSCs in culture (Figure 1A). We screened 92 biologically-active natural products collected from commercially available compounds (Table S1), and identified Garcinol (GAR) as one of the most active compounds (Figure 1B and C). To evaluate the function of GAR in detail and estimate the structure-activity relationship, we synthesized its derivatives, Isogarcinol (ISO), O-monomethylisogarcinol (MMI), and O-dimethylisogarcinol (DMI) (Figure 1B). We then cultured hCB CD34^+^ cells in medium supplemented with SCF, TPO and the Garcinol derivatives for 7 days. GAR, ISO, and MMI facilitated the expansion of CD34^+^CD38^–^ cells compared with the DMSO control (Figure 2A), but little affected the total cell numbers at their effective concentrations (10 µM of GAR: 109.7±10.3%, 5 µM of ISO: 71.5± 23.7%, 2 µM of MMI: 91.1± 2.5%, 0.5 µM of DMI: 93.0±4.1% relative to the blank control). We observed a more efficient effect by GAR, ISO, and MMI when hCB CD34^+^CD38^–^ cells were used as the starting material (Figure 2B). During the 7-day culture, CD34^+^CD38^-^ cells expanded in number 4.5, 7.4, 2.2, and 1.4-fold with GAR, ISO, MMI, and DMI, respectively, as compared with the blank culture. These results indicated that GAR derivatives other than DMI increased the number of primitive CD34^+^CD38^-^ cells efficiently in culture.

To evaluate the number of functional HSCs/HPCs in cultures with GAR derivatives, we next performed colony assays. Cultured cells with GAR derivatives contained all types of myeloid progenitors, and there was no significant difference in total colony number between the culture with GAR derivatives and blank. In contrast, high proliferative potential colony-forming cells (HPP-CFCs), which give rise to colonies with a diameter greater than 1 mm, [22] and colony-forming units-granulocyte/erythrocyte/macrophage/megakaryocyte (CFU-GEMM), which represent the most primitive progenitors, were more frequently contained in the cultures with GAR, ISO, and MMI than those with DMSO (Figure 2C). As with the effect on CD34^+^CD38^-^ cell proliferation in culture, DMI showed no activity to expand HPP-CFC and CFU-GEMM numbers (Figure 2A-C). In all combinations of cytokines tested, the addition of GAR further increased the numbers of CD34^+^ and CD34^+^CD38^-^ cells compared to each blank control, even in the presence of SCF, TPO, and FL, the most potent cytokine combination tested (Figure 2D). These results showed that GAR derivatives other than DMI had the positive effect on the primitive hematopoietic progenitor proliferation during the 7-day culture.

### GAR-treated CD34^+^ cells include increased numbers of SRCs

**Figure 3:**
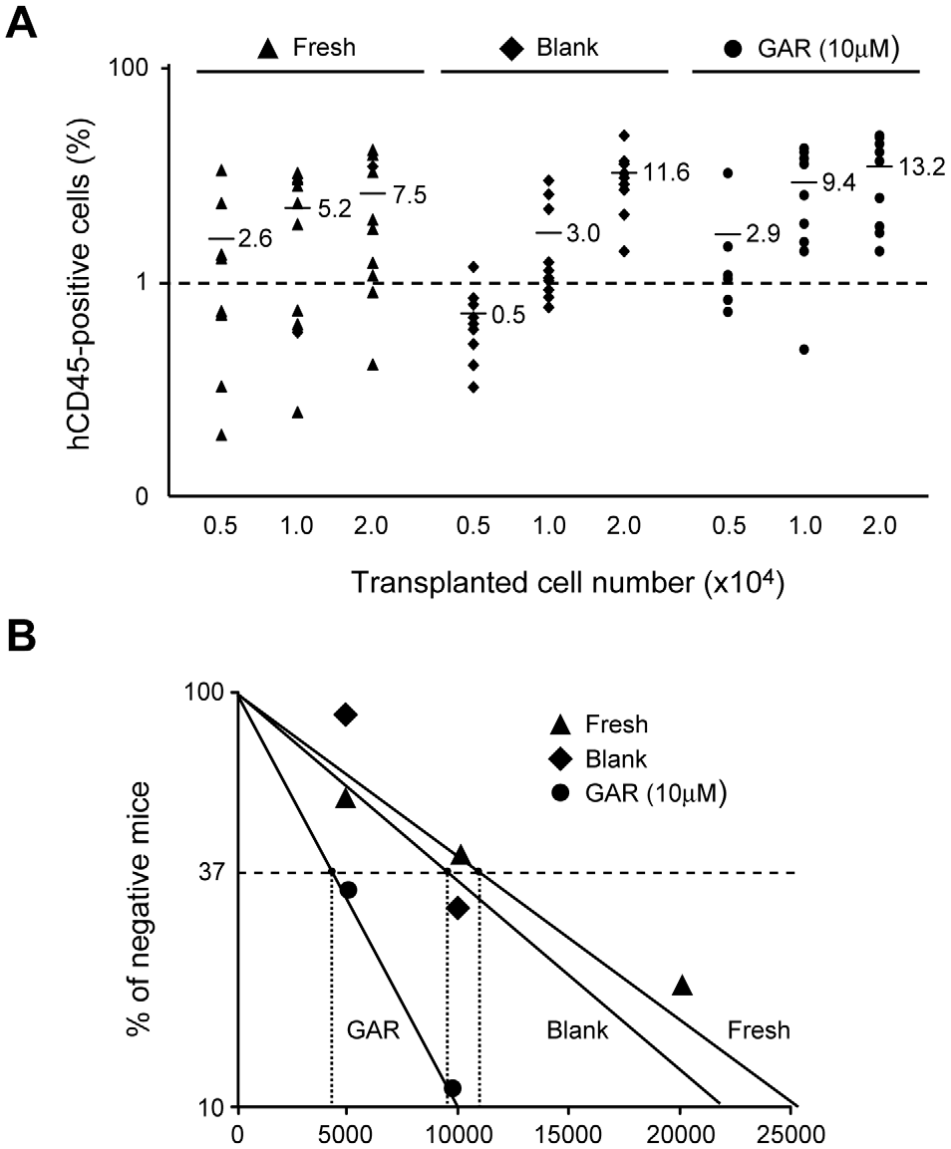
Garcinol efficiently expands SRC numbers. **A.** hCB CD34^+^ cells were cultured with 10 µM of GAR in the presence of rhSCF, rhTPO and rhFL for 7 days. NOD/SCID mice (n = 91) were injected with increasingly higher doses (5×10^3^, 1×10^4^, 2×10^4^ cells/mice) of freshly isolated hCB CD34^+^ cells, cells cultured with DMSO (blank), or cells cultured with GAR, and the chimerism of human CD45^+^ cells in the recipient BM cells was analyzed 8 weeks after transplantation. Bars represent the mean values of chimerism. **B.** Mice with at least 1% human CD45^+^ cells in BM in A were considered successfully engrafted and the frequency of SRCs was determined with L-Calc software (StemCell Technologies).

To evaluate the number of functional HSCs in cultures with GAR, we performed a NOD/SCID-repopulation assay [19], [23] to estimate the capacity for reconstitution among the progeny of hCB CD34^+^ cells cultured with GAR in addition to SCF, TPO, and FL. We used limiting numbers of cells for the repopulation assay to estimate the SRC frequencies. Increasing numbers (5×10^3^, 1×10^4^, 2×10^4^) of fresh CD34^+^ cells or 7-day cultured cells corresponding to the same number of input CD34^+^ cells were transplanted into NOD/SCID mice. The average repopulation levels by human hematopoietic CD45^+^ cells were higher in recipient mice infused with GAR-treated cells than in those infused with fresh CD34^+^ cells or vehicle (DMSO)-treated cells (Fig. 3A). The frequency for SRCs was 1 in 10,921 (95% confidence interval of 1 of 14,109 to 1 of 8,453) among fresh CD34^+^ cells and 1 in 9,521 (95% confidence interval of 1 of 12,318 to 1 of 7,358) in the culture with vehicle (DMSO). In contrast, the frequency for SRCs in the culture with GAR was 1 in 4,328 (95% confidence interval of 1 of 5,844 to 1 of 3,206), which was 2.5-fold higher than for fresh CD34^+^ cells (p = 0.019) and 2.2-fold higher than for the vehicle cultures (p = 0.046) (Fig. 3B). These results demonstrated that GAR promoted the expansion of SRCs in culture.

It has been reported that expression of homing receptors is critical for hCB SRCs to efficiently engraft bone marrow (BM) [24]. Therefore, we evaluated the levels of surface CD184 (CXCR4) and CD62L (L-selectin) on CD34^+^ cells cultured for 7 days with or without GAR in the presence of SCF, TPO, and FL. As compared with the blank culture, the expression levels of homing receptors on GAR-treated CD34^+^ cells were comparable or slightly decreased; the population of CD34^+^CD184^+^ cells was 1.6±0.3% [mean fluorescence intensity (MFI); 35.5] and 1.0±0.2% (MFI; 38.2) in control and GAR cultures, respectively, and the population of CD34^+^CD62L^+^ cells was 3.7±0.6% (MFI; 28.5) and 3.1±0.2% (MFI; 26.8) in control and GAR cultures, respectively (n = 3). These data indicated that the enhanced engraftment by GAR-treated CD34^+^ cells is attributed to an increase in SRC number rather than augmented homing capacity during culture.

### GAR inhibits HAT activity and protein acetylation

**Figure 4:**
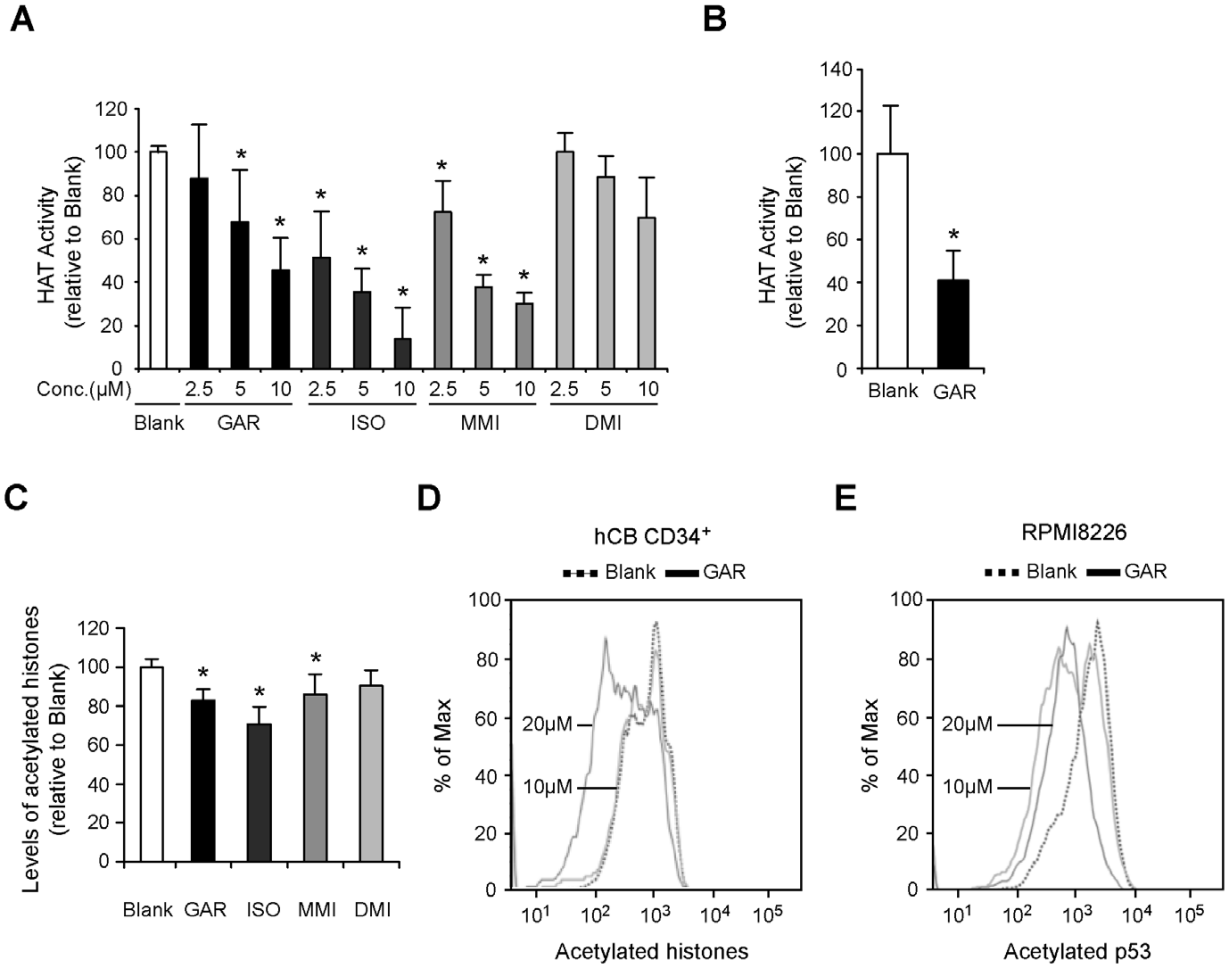
Garcinol inhibits histone acetyltransferase activity. **A.** Garcinol functions as an inhibitor of HAT. Nuclear extracts from HL60 cells (1×10^6^) cultured with the indicated concentrations of Garcinol derivatives or the same volume of DMSO (blank) for 24 h were assayed for HAT activity. Bars represent the relative activity of HAT (mean±SEM, n = 3−5). *p<0.05. **B.** Garcinol inhibits the HAT activity of hCB CD34^+^ cells. hCB CD34^+^ cells (5×10^5^) were cultured with 10 µM of GAR or the same volume of DMSO (blank) in the presence of 20 ng/mL of hrTPO and 100 ng/mL of hrSCF for 3 days, and the nuclear extracts of cultured cells were assayed for HAT activity. Bars represent the relative activity of HAT (mean±SEM, n = 5). *p<0.05. **C.** Garcinol reduces the levels of histone acetylation. HeLa cells (2×10^4^) were cultured with 10 µM of Garcinol derivatives or the same volume of DMSO (blank) for 24 h. The levels of histone acetylation in the cultured cells were measured by using an EpiQuik HAT activity/inhibition assay kit. Bars represent the levels of acetylated histones relative to the blank (mean±SEM, n = 3). *p<0.05. **D.** Garcinol reduces the levels of histone acetylation in hCB CD34^+^ cells. hCB CD34^+^ cells (5×10^5^) were cultured with 10 or 20 µM of GAR or the same volume of DMSO (blank) for 3 days. The cultured cells were fixed and permeabilized and then stained with Alexa Fluor 488-conjugated anti-acetylated histone H3 antibody. Flow cytometric analysis was performed to measure the levels of histone acetylation. Data are representative of 3 independent experiments. **E.** Garcinol inhibits acetylation of p53 at K382. RPMI8226 cells (1×10^6^) were cultured with 10 or 20 µM of GAR or the same volume of DMSO (blank) for 1 day. The levels of acetylation of p53 at K382 were measured by using Alexa Fluor 647-conjugated mouse anti-p53/acK382 antibody. Data are representative of 3 independent experiments.

GAR has been characterized as a non-specific HAT inhibitor and its derivative, ISO, was demonstrated to inhibit both p300 and PCAF [21]. To confirm the reported effect of GAR derivatives on intracellular HAT activity, we observed HAT activity in cells treated with GAR derivatives. Indeed, treatment of cells with GAR, ISO, and MMI, but not DMI, inhibited cellular HAT activity in HL60 cells (Figure 4A). The inhibitory effect of GAR and its derivatives on HAT correlated well with their capacity to expand HSCs/PCs *ex vivo*. GAR also inhibited HAT activity in hCB CD34^+^ cells (Figure 4B). Next, we observed the level of protein acetylation, which is regulated by HATs, in cells treated with GAR derivatives. In accordance with the result of HAT inhibition, GAR, ISO, and MMI, but not DMI, reduced cellular histone acetylation levels in HeLa cells (Figure 4C). A reduced level of acetylation was also observed in hCB CD34^+^ cells treated with GAR (Figure 4D). As with histone acetylation, GAR reduced the level of acetylation of p53 at K382 in RPMI8226 cells (Figure 4E).

### GAR-treated cells show unique profiles of gene expression

**Figure 5:**
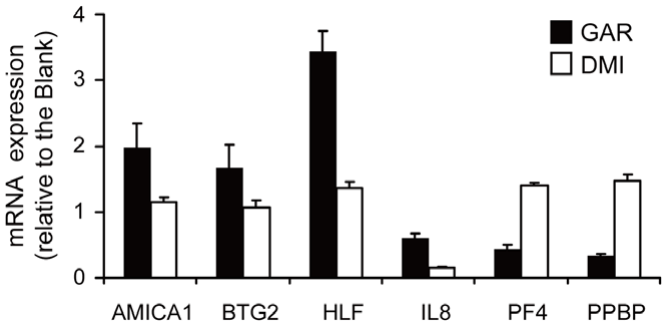
Treatment of GAR modifies the gene expression in CD34^+^CD38^–^ cells. hCB CD34^+^ cells were cultured with 10 µM of GAR or 0.5 mM of DMI in the presence of rhSCF, rhTPO and rhFL for 7 days. Bars represent the mean of fold-changes in gene expression relative to the blank (n = 3−5) detected by quantitative real-time PCR. The *peptidylprolyl isomerase A* or *beta-2-microglobulin* gene was used as an internal control.

To understand the molecular mechanisms by which GAR promotes the expansion of HSCs, we examined global transcription levels of genes in cultured CD34^+^CD38^–^ cells with GAR using a DNA microarray. Treatment of HSCs/PCs with GAR for 7 days led to the up-regulation of 20 genes and down-regulation of 9 genes in CD34^+^CD38^–^ cells (Table 1). Among them, we validated the expression of 6 genes by using real-time quantitative PCR (Figure 5). Treatment of HSCs/PCs with GAR resulted in a 1.98, 1.67, and 3.44-fold increase in *AMICA1*, *BTG2* and *HLF* expression, respectively, and a 0.61, 0.43, and 0.33-fold decrease in *IL8*, *PF4*, and *PPBP* expression, respectively, in the CD34^+^CD38^–^ cell fraction. In contrast, DMI did not significantly change the expression of these genes, except for *IL8*. The transcriptional levels of other genes reported to be implicated in the self-renewal of HSCs, for example, *HOXB4*, *BMI1*, *GATA2*, *NOTCH1*, *p21*, *p27*, *c-MYC*, *EGR*, and *EVI-1* [25], [26], were not changed by GAR (data not shown).

**Table 1:** Gene expression changes in GAR-treated cells.

Symbol	Gene	Fold-change
ADRB1	adrenergic, beta-1-, receptor	2.6
AMICA1	adhesion molecule, interacts with CXADR antigen1	1.5
BTG2	BTG family, member2	1.8
CNTNAP5	contactin associated protein-like5	7.3
COL5A1	collagen, type V, alpha 1	1.6
CYP1B1	cytochrome P450, family 1, subfamily B, polypeptide 1	1.9
FAM55C	family with sequence similarity 55, member C	2.2
FBXL17	F-box and leucine-rich repeat protein 17	1.7
HLF	hepatic leukemia factor	3.1
IGJ	immunoglobulin J polypeptide	3.0
JAG2	jagged 2	1.9
LRBA	LPS-responsive vesicle trafficking, beach and anchor containing	2.7
NUPL1	nucleoporin like 1	3.2
PTK2	PTK2 protein tyrosine kinase 2	2.2
SEMA3C	sema domain, immunoglobulin domain (Ig)	2.3
SLC41A2	solute carrier family 41, member 2	3.2
THAP9	THAP domain containing 9	2.0
TRIM59	tripartite motif-containing 59	1.6
ZNF407	zinc finger protein 407	6.3
ZNF623	zinc finger protein 623	2.1
CST7	cystatin F (leukocystatin)	0.7
HBD	hemoglobin, delta	0.6
IL8	interleukin 8	0.7
INPP5F	inositol polyphosphate-5-phosphatase F	0.7
PF4	platelet factor 4	0.6
PPBP	pro-platelet basic protein	0.6
RPS2	ribosomal protein S2	0.6
TP73	tumor protein p73	0.6
TP53I11	tumor protein p53 inducible protein 11	0.4

The gene expression analysis was performed using total RNA of CD34^+^CD38^–^ cells sorted from 7-day cultures of hCB CD34^+^ cells with 10 µM of GAR, 0.5 µM of DMI, or the same volume of DMSO (blank). The DNA microarray assay was conducted with a GeneChip 3′expression array (Kurabo, Osaka, Japan). The genes whose expression was up-regulated greater than 1.5-fold or down-regulated less than 0.7-fold upon treatment with GAR compared to the blank control are shown.

## Discussion

A search for biologically active natural products that promote HSC expansion uncovered GAR, a benzophenone derivative originally isolated from Garcinia indica [20], [21]. GAR is the first plant-derived natural product found to act on HSCs/PCs. Isogarcinol, a derivative of GAR, was even more active, further supporting the efficacy of GAR. As reported before, GAR exerted its activity as a HAT inhibitor in HSCs/PCs. GAR reduced cellular HAT activity and the levels of histone acetylation in hCB CD34^+^ cells. Importantly, the inhibitory effect of GAR and its derivatives on HAT correlated well with their capacity to expand HSCs/PCs *ex vivo*. Together, our findings provide the first evidence of the effectiveness of inhibiting HAT on the expansion of HSCs/PCs. GAR also reduced the level of acetylation of p53 at K382 in RPMI8226 cells, although its effect was not obvious in hCB CD34^+^ cells (data not shown). p53 restricts the self-renewal of HSCs: *p53*-deficient mice have been reported to contain twice as many HSCs and their HSCs exhibit greater repopulating capacity than do wild-type HSCs [27]. p300 HAT is reported to acetylate p53 at K382, and, by doing so, enhances the sequence-specific DNA binding of p53 [28]. Thus, GAR might expand HSCs/PCs, at least partly, through suppression of p53 activity.

Several SMCs have also been reported to be effective in the manipulation of HSCs/PCs by inhibiting components of the epigenetic machinery. Araki et al. proposed that the treatment of hCB HSCs with a DNA methyltransferase inhibitor, 5-aza-2′-deoxycytidine, followed by a histone deacetylase (HDAC) inhibitor, trichostatin A, efficiently expands HSCs [29], [30]. However, inhibitors for DNA methyltransferases or HDACs are also effective against cancer cells and restrict their tumorigenic activity by releasing the transcriptional repression of tumor suppressor genes [31]. So, their efficacy in normal HSCs/PCs might need further evaluation. By contrast, we previously reported that forced expression of *Bmi1*, one of the polycomb-group genes, augments stem cell activity [32]. The polycomb-group proteins transcriptionally repress tumor suppressor genes by adding repressive histone modifications, the trimethylation of histone H3 at lysine 27 and monoubiquitylation of histone H2A at lysine 119, to their promoters [33], [34]. Forced expression of *Bmi1* reinforces the transcriptionally repressive state of tumor suppressor genes, such as *Ink4a* and *Arf*. We speculate that GAR acts in a similar fashion to Bmi1 by inhibiting the activity of HATs. Correspondingly, not many genes were altered in their expression by GAR. Given the broad effects of GAR on cellular acetylation levels, the limited effect on gene expression was unexpected, but may suggest a specific function of GAR in maintenance of the transcriptionally repressive state of tumor suppressor genes. Nevertheless, the up-regulated gene expression of hepatic leukemia factor (HLF), a member of the proline and acidic-rich protein family, is intriguing because HLF has been implicated in the control of human HSC function: Forced expression of HLF in human HSCs reportedly protects against apoptosis and enhances *in vivo* reconstitution [35].

In summary, GAR and its derivatives modulate the acetylation of not only histones but also key HSC regulators such as p53 to establish a gene expression profile and molecular functions favorable for HSC/PC expansion. Thus, the inhibition of HAT represents a new approach to HSC manipulation and therapy. It is important to find out more potent, specific, and less toxic HAT inhibitors to precisely revalidate their effectiveness on HSCs/PCs and apply them to the manipulation of HSCs/PCs *ex vivo*. Commercially available HAT inhibitors, curcumin [36] and anacardic acid [37], were toxic and less active on HSCs/PCs compared with GAR (data not shown). Additional screening of HAT inhibitors might be required to obtain suitable compounds for the expansion of HSCs/PCs *ex vivo*. Of interest, GAR did not have any antagonistic function against aryl hydrocarbon, and thus exerted an additive effect on the expansion of HSCs/PCs in combination with SR-1, an antagonist of aryl hydrocarbon receptor [16] (data not shown). These findings suggest that the combination of SMCs with different molecular targets would improve the efficacy of HSC/PC expansion *ex vivo*.

## Materials and Methods

### Ethics Statement

All experiments using the mice were performed in accordance with our institutional guidelines for the use of laboratory animals and approved by the review board for animal experiments of Chiba University (approval ID: 21–150). The study using hCB cells was approved by the institutional ethics committees of Chiba University (approval ID: 692).

### Reagents

Natural products and chemical derivatives for screening were collected from the library LOPAC^1280^ (Sigma-Aldrich, Missouri) or purchased from Cosmo Bio (Tokyo, Japan). GAR was obtained from Enzo Life Sciences (New York, USA) and its derivatives were chemically synthesized as reported previously [21]. Briefly, GAR was stirred for 10 hours in toluene and hydrochloric acid at room temperature, and left for 17 hours at 4°C. The reaction mixture was then filtered, and the residue was washed with distilled water and acetonitrile. Isogarcinol (ISO) was finally obtained by recrystallization in acetonitrile. ISO was then methylated by addition of potassium carbonate and methyl iodide for 17 hours at 25°C. The product was extracted with ethyl acetate and concentrated. O-monomethylisogarcinol (MMI) and O-dimethylisogarcinol (DMI) were separated by silica gel column chromatography. Yeild: ISO; 46% (68.7 mg), MMI; 38% (17.7 mg), and DMI; 88% (27.7 mg).

### Mice

Nonobese diabetic/severe combined immunodeficient (NOD/SCID) mice were purchased from CLEA Japan (Tokyo, Japan).

### Cell culture

Human cord blood (hCB) CD34^+^ cells were purchased from Lonza (Basel, Switzerland) or purified from hCB obtained from the Tokyo Cord Blood Bank (Tokyo, Japan). Mononuclear cells were separated by density gradient centrifugation. CD34^+^ cells were immunomagnetically enriched using a magnetic-activated cell sorting CD34 progenitor kit (Miltenyi Biotech, California). The purity of hCB CD34^+^ cells was over 95%. CD34^+^CD38^–^ cells were isolated by fluorescence-activated cell sorting using a BD FACSAria (BD Bioscience, California). Purified CD34^+^ and CD34^+^CD38^–^ cells were cryopreserved or used immediately for experiments. hCB CD34^+^ and CD34^+^CD38^–^ cells were plated at 1×10^4^ cells/well in a 24-well plate precoated with 25 µg/mL of fibronectin fragment CH-296 (Takara Shuzo, Otsu, Japan) [17] and cultured in serum-free medium, StemSpan SFEM (Stem Cell Technologies, Vancouver, Canada) supplemented with a 1% penicillin-streptomycin mixture (Sigma) at 37°C in a humidified atmosphere flushed with 5% CO2 in air. Recombinant human (rh)SCF (Wako Pure Chemical Industries, Osaka, Japan) was added at 100 ng/ml, rhFL (PeproTech, New Jersey) at 50 ng/mL, and rhTPO (PeproTech) at 20 ng/mL. Garcinol derivatives were added in the indicated amounts. Human leukemia cell lines, HL60 [38] and RPMI8226 [39], were purchased from DS Pharma Biomedical (Osaka, Japan) and cultured in RPMI 1640 medium (Invitrogen, California) containing 10% fetal bovine serum and a 1% penicillin-streptomycin mixture (Sigma) at 37°C under 5% CO2 in a humidified incubator and passaged every three days. The human epithelial carcinoma cell line HeLa was cultured in Dulbecco's modified Eagle's medium (DMEM) supplemented with 10% fetal bovine serum and a 1% penicillin-streptomycin mixture (Sigma).

### Flow cytometry

Cultured hCB CD34^+^ cells were stained with allophycocyanin (APC)-conjugated anti-human CD34 and phycoerythrin (PE)-conjugated anti-human CD38 antibodies (BD Pharmingen, California). Then 1 µg/mL of propidium iodide (Sigma) was added to exclude nonviable cells. Cells were analyzed on a BD FACSCant II (BD Bioscience) or a JSAN desktop cell sorter (Bay Bioscience, Kobe, Japan). For analyzing hematopoietic engraftment in NOD/SCID mice, BM cells were stained with APC-conjugated CD45 antibody (BD Pharmingen). To measure the acetylated levels of histones and p53 protein, cultured cells were fixed and permeabilized with a Cytofix/Cytoperm Fixation/Permeabilization Solution Kit (BD Biosciences) and then stained with Alexa Fluor 488-conjugated anti-acetylated histone H3 antibody (clone C5B11, Cell Signaling) and Alexa Fluor 647-conjugated mouse anti-p53/acK382 antibody (clone L82-51, BD Biosciences), respectively. To measure the expression levels of homing receptors on cell surface, cultured hCB CD34^+^ cells were stained with APC-conjugated anti-human CD34, PE-conjugated anti-human CD184 (clone 12G5. BD Pharmingen), and fluorescein isothiocyanate-conjugated anti-human CD62L antibodies (clone DREG-56, BD Pharmingen) and analyzed on a JSAN desktop cell sorter.

### Colony forming assay

hCB CD34^+^ cells, which were cultured with Garcinol for 7 days, were plated in Methocult GF H4435 methylcellulose medium containing 50 ng/mL human SCF, 10 ng/mL human granulocyte-macrophage colony-stimulating factor, 10 ng/mL human IL-3, and 3 U/mL human EPO (StemCell Technologies). After 12 to 14 days of culture, the colonies were counted under a microscope.

### Histone acetylation assay

The histone acetylation activity in nuclear extracts and the levels of acetylated lysine in test cells were evaluated using an EpiQuik HAT activity/inhibition assay kit (Epigentek, Brooklyn, NY) and Cellular histone acetylation assay kit (CycLex, Nagoya, Japan), respectively.

### Gene expression analysis

hCB CD34^+^ cells were cultured in the presence of Garcinol derivatives or DMSO for 7 days, and then CD34^+^CD38^–^ cells were isolated by cell sorting with a FACSAria (BD Bioscience). Total RNA of the isolated cells was extracted using an RNeasy Mini kit (Qiagen, California). Gene expression was analyzed with a GeneChip 3′expression array (Kurabo, Oosaka, Japan). All data is MIAME compliant and that the raw data was deposited in Gene Expression Omnibus (accession number GSE29459). The total RNA (1 µg) was reverse-transcribed with a SuperScript one-step RT-PCR kit (Invitrogen). Real-time PCR was carried out for 40 to 45 cycles of 1 minute at 60°C and 15 seconds at 95°C in an ABI PRISM 7700 Sequence Detector (Applied Biosystems, CA). All Taqman primers and probes were obtained from Applied Biosystems.

### Transplantation of hematopoietic cells into NOD/SCID mice

NOD/SCID mice at 8–10 weeks of age were sublethally irradiated with a dose of 2.75 Gy. Fresh and cultured hCB CD34^+^ cells were injected intravenously. At 8 weeks post-transplantation, the mice were sacrificed and bone marrow (BM) cells were analyzed with a JSAN desktop cell sorter (Bay Bioscience) for the presence of human CD45^+^ cells. To obtain the frequency of SRCs, assays were performed using limiting doses of test cells and the data were analyzed using L-Calc software (StemCell Technologies).

### Statistical analysis

All results are presented as the mean±standard error of the mean (SEM). Statistical significance was analyzed with Student's t-test or Williams' test. The level of significance was set at 0.05.

## Supporting Information

Table S1 **List of natural products screened.**
(DOC)
