# Supplementary material for: Ex Vivo Expansion of Human Hematopoietic Stem Cells by Garcinol, a Potent Inhibitor of Histone Acetyltransferase
Source: PLoS One. 2011 Sep 12;6(9):e24298. doi: 10.1371/journal.pone.0024298 (PMC3171405; doi:10.1371/journal.pone.0024298)
Supplement: Table S1 — List of natural products screened. (DOC) [file pone.0024298.s001.doc]

**Table S1. List of natural products screened.**

| Actinonin |
| --- |
| Aconitine |
| Anacardic acid |
| 4-Androsten-4-ol- 3,17-dione |
| N-arachidonylglycine |
| Astaxanthin |
| 5-azacytidine |
| 3'-Azido-3'-deoxythymidine |
| N6-2-(4-Aminophenyl)ethyladenosine Adenosine |
| Ap4A |
| Beclomethasone |
| Betamethasone |
| Bestatin hydrochloride |
| Brefeldin A |
| (+)-Bromocriptine methanesulfonate |
| (E)-5-(2-Bromovinyl)-2'-deoxyuridine |
| 8-Bromo-cAMP sodium |
| 8-Bromo-cGMP sodium |
| 5-Bromo-2'-deoxyuridine |
| (±)-Butaclamol hydrochloride |
| Budesonide |
| Butein |
| Butaclamol hydrochloride |
| Bromoacetyl alprenolol menthane |
| Cantharidin |
| (+)-Catechin Hydrate |
| Cephalexin hydrate |
| Cefazolin sodium |
| Cefaclor |
| Ceftriaxone sodium |
| Cefmetazole sodium |
| Cefotaxime sodium |
| Cephalosporin C zinc salt |
| Cephalothin sodium |
| (-)-Cotinine |
| Cephradine |
| Cheatocin  Corticosterone |
| Cortisone |
| CP55940 |
| Cyclosporin A |
| Cefsulodin sodium salt hydrate |
| 2-Chloroadenosine |
| Cyproterone acetate |
| N6-Cyclopentyladenosine |
| Colchicine |
| Cytosine-1-beta-D-arabinofuranoside hydrochloride |
| Calcimycin |
| Curcumine |
| Cantharidic Acid |
| L-Canavanine sulfate |
| Garcinol |
| (±)-3-(2-Carboxypiperazin-4-yl)propyl-1-phosphonic acid |
| 2-Chloro-N6-(3-iodobenzyl)-adenosine-5'-N-methyluronamide |
| 8-(4-Chlorophenylthio)-cAMP sodium |
| beta-Chloro-L-alanine hydrochloride |
| Cortisone 21-acetate |
| 2-Chloro-2-deoxy-D-glucose |
| (S)-(+)-Camptothecin |
| 2-Chloroadenosine triphosphate tetrasodium |
| D-Cycloserine |
| Capsazepine |
| Dihydrokainic acid |
| Dihydroergotamine methanesulfonate |
| 2',3'-didehydro-3'-deoxythymidine |
| 2',3'-dideoxycytidine |
| 1,4-Dideoxy-1,4-imino-D-arabinitol |
| 5'-diphosphate trisodium salt hydrate |
| 2,4-Dinitrophenyl 2-fluoro-2-deoxy-beta-D-glucopyranoside |
| D-ribofuranosylbenzimidazole |
| Fulvestrant |
| Gabaculine hydrochloride |
| gamma-Aminobutyric acid |
| Ganaxolone |
| GR 79236X |
| Imipenem monohydrate |
| L-3,4-Dihydroxyphenylalanine methyl ester hydrochloride |
| Methotrexate hydrate |
| 2-Methylthioadenosine triphosphate tetrasodium |
| 2-(Methylthio) adenosine |
| (-)-alpha-Methylnorepinephrine |
| N-Methyl-1-deoxynojirimycin |
| Nipecotic acid |
| NG,NG-Dimethylarginine hydrochloride |
| N6-Cyclohexyladenosine |
| N6-(4-Aminobenzyl)-9-[5-(methylcarbonyl)-beta-D-ribofuranosyl] adenine |
| O6-benzylguanine |
| Reserpine |
| Sodium Taurocholate hydrate |
| SU5402 |
| trans-(±)-1-Amino-1,3-cyclopentanedicarboxylic acid |
| Trichostatin A |
| Z-L-Phe chloromethyl ketone |
| (2S,1'S,2'S)-2-(carboxycyclopropyl)glycine |
| Valporic acid |
| Zebularine |
